# Sub-Nucleolar Trafficking of Hendra Virus Matrix Protein Is Regulated by Ubiquitination

**DOI:** 10.3390/v17060797

**Published:** 2025-05-30

**Authors:** Tianyue Zhao, Florian A. Gomez, Cassandra T. David, Christina L. Rootes, Cameron R. Stewart, Gregory W. Moseley, Stephen M. Rawlinson

**Affiliations:** 1 Department of Microbiology, Biomedicine Discovery Institute, Monash University, 19 Innovation Walk, Clayton Campus, Clayton, VIC 3800, Australia; 2 Commonwealth Scientific and Industrial Research Organisation (CSIRO), Health and Biosecurity, Australian Centre for Disease Preparedness, Geelong, VIC 3219, Australia

**Keywords:** Hendra virus, matrix, nucleolus, henipavirus, trafficking, ubiquitination

## Abstract

Hendra virus (HeV) is a highly pathogenic member of the *Henipavirus* genus (family *Paramyxoviridae*, order *Mononegavirales*), for which all basic replication processes are located in the cytoplasm. The HeV matrix (M) protein plays essential roles in viral assembly and budding at the plasma membrane, but also undergoes dynamic nuclear and nucleolar trafficking, accumulating in nucleoli early in infection, before relocalising to the plasma membrane. We previously showed that M targets sub-nucleolar compartments—the fibrillar centre (FC) and dense fibrillar component (DFC)—to modulate rRNA biogenesis by mimicking a process occurring during a nucleolar DNA-damage response (DDR). Here, we show that M protein sub-nucleolar localisation is regulated by ubiquitination, which controls its redistribution between the FC-DFC and granular component (GC). The mutagenesis of a conserved lysine (K258) reported to undergo ubiquitination, combined with the pharmacological modulation of ubiquitination, indicated that a positive charge at K258 is required for M localisation to the FC-DFC, while ubiquitination regulates subsequent egress from the FC-DFC to the GC. M proteins from multiple *Henipaviruses* exhibited similar ubiquitin-dependent sub-nucleolar trafficking, indicating a conserved mechanism. These findings reveal a novel mechanism regulating viral protein transport between phase-separated sub-nucleolar compartments and highlight ubiquitination as a key modulator of intra-nucleolar trafficking.

## 1. Introduction

**Figure 1:**
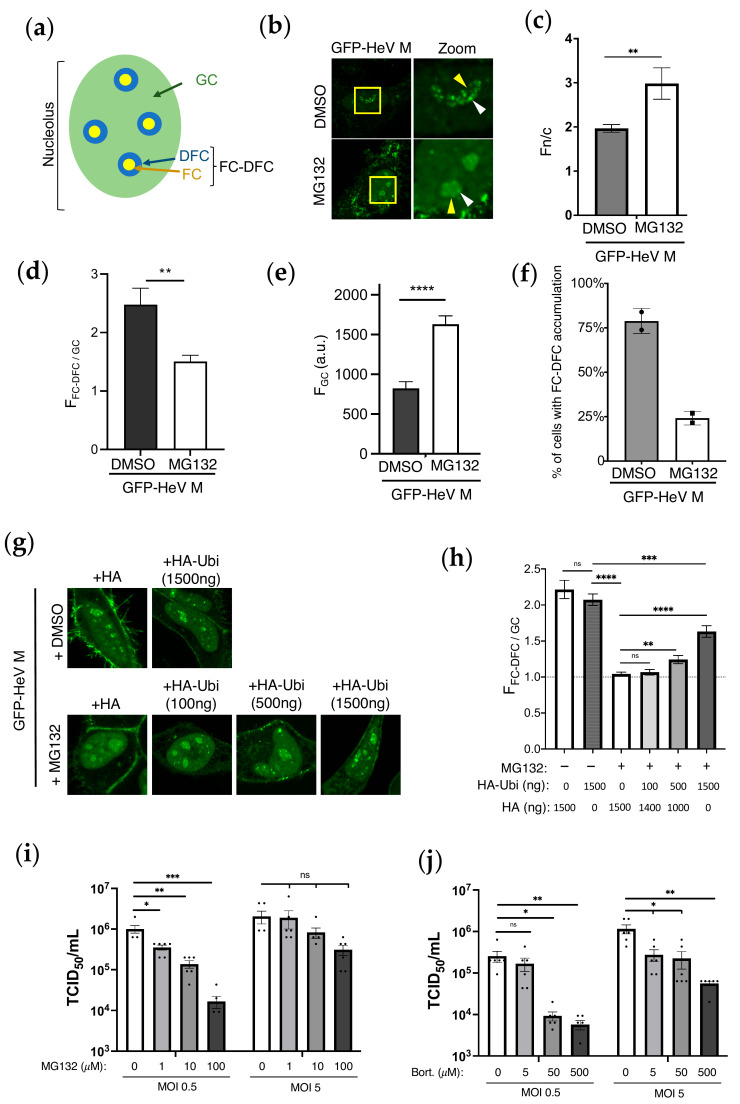
**Ubiquitination regulates FC-DFC accumulation of HeV M and impacts on virus production.** (**a**) Schematic of a nucleolus showing the three primary sub-compartments: fibrillar centre (FC), dense fibrillar component (DFC), and granular component (GC). The region composed of the FC and DFC compartments is referred to as the FC-DFC. (**b**) HeLa cells transfected to express GFP-HeV M protein were treated 18 h post-transfection (p.t.) with MG132 or without (DMSO) for 6 h before CLSM analysis. Representative images are shown for each condition; yellow boxes are magnified in the zoom panel. Yellow arrowheads indicate nucleoli; white arrowheads indicate localisation of M protein to sub-nucleolar compartments consistent with FC-DFC. Images such as those in B were analysed to determine the following: (**c**) the nuclear to cytoplasmic (Fn/c) fluorescence ratio; (**d**) the ratio of fluorescence of the FC-DFC to that of the GC (F_FC-DFC/GC_); (**e**) the fluorescence intensity of the GC (F_GC_) (arbitrary units (a.u.)); and (**f**) the % of HeV M-expressing cells with apparent accumulation in FC-DFC (histogram shows the percentage of M protein-expressing cells containing at least one nucleolus with evident accumulation of M protein into one or more FC-DFC). Histograms for C, D, and E show mean ± S.E.M., *n* ≥ 24 cells for each condition (data from a single assay, consistent with two independent experiments); histogram in F shows mean percentage ± SD from two independent assays, *n* ≥ 73 cells for each condition. (**g**) HeLa cells co-transfected with plasmid to express GFP-HeV M and with differing amounts of HA or HA-ubiquitin (HA-Ubi) expression plasmid (1500 ng total HA/HA-Ubi plasmid transfected, comprising HA-Ubi and/or HA, as indicated) and treated without (DMSO) or with MG132. (**h**) Images such as those in G were used to calculate the F_FC-DFC/GC_. Data from a single assay (*n* = 24 cells per sample), representative of two independent assays. Dashed line indicates F_FC-DFC/GC_ of 1. (**i**,**j**) HeLa cells infected with HeV at MOI 0.5 or MOI 5 and treated with increasing concentrations of proteosome inhibitors, (**i**) MG132 or (**j**) Bortezomib (Bort), prior to collection at 42 h p.i. and determination of HeV titres (TCID50/mL ± S.E.M., *n* = 6). Statistical analysis used Student’s *t*-test; * *p* < 0.05; ** *p* < 0.01; *** *p* < 0.001; **** *p* < 0.0001; ns, non-significant.

The nucleolus comprises a highly multifunctional structure with long established roles in ribosome biogenesis, as well as roles in cell cycle regulation, the DNA damage response (DDR), cellular stress responses, and signal recognition particle assembly [1,2]. The nucleolus was recently shown to be a membrane-less organelle (MLO) comprising at least three immiscible liquid condensates that are formed by liquid–liquid phase separation (LLPS) [3]. The three components are the fibrillar centre (FC), dense fibrillar component (DFC), and granular component (GC). The FC is surrounded by the DFC to form functional units (FC-DFC), which are embedded within the GC (Figure 1a and Figure S1) [4]. These compartments play distinct roles, including assembling a pipeline for the key steps of ribosome biogenesis.

Consistent with its multifunctionality, the nucleolus is a common target of diverse viruses [5,6,7]. This targeting is proposed to enable the viral exploitation of diverse processes to usurp host cell biology and/or facilitate virus replication [5,6,7]. Despite the prevalence of viral protein nucleolar targeting, functional outcomes generally remain poorly understood. The potential nucleolar functions of viral proteins are of particular interest with respect to RNA viruses that typically have limited coding capacity and replicate their genomes in the cytoplasm, but nevertheless target specific proteins to nucleoli. These include the highly pathogenic non-segmented negative sense RNA viruses (nsNSVs), which includes the Hendra (HeV) and Nipah (NiV) viruses (genus *Henipavirus*, family *Paramyxoviridae*), the matrix (M) proteins of which localises to the nucleus and nucleolus during infection [8,9,10,11].

Henipavirus M proteins play critical roles in virus assembly in the cytoplasm and in budding at the plasma membrane [12,13]. The subcellular localisation of M protein is dynamic, being nucleolar early in infection before exiting the nucleolus/nucleus and accumulating at the plasma membrane for assembly and budding [8,9,10,11]. Interestingly, transit through the nucleolus is reported to be a prerequisite for M protein to fulfill assembly and budding functions, suggestive of a regulatory role of nucleoli in viral release [10,11]. Genetic screens have indicated the importance of nucleolar proteins in infection, and proteomic datasets suggest that M proteins interact with multiple nucleolar proteins [8,10,14,15]. However, the potential intra-nucleolar roles of HeV M protein remained unresolved until the identification of a novel nucleolar function whereby HeV M localises to a sub-nucleolar compartment corresponding to the FC-DFC, where it interacts with Treacle protein and impairs ribosomal RNA (rRNA) biogenesis [14]. This process appears to be mediated by the mimicry of a cellular process that normally occurs during a DDR. Thus, subcellular trafficking underpins key functions of HeV M. However, how this trafficking is regulated, particularly between sub-nucleolar compartments and other regions of the cell, remains unresolved. Indeed, the mechanisms regulating the trafficking of proteins in general between sub-nucleolar liquid condensates is poorly understood, with no prior studies to our knowledge, for any viral protein.

Previously, we showed that the substitution of residue K258 in HeV M for alanine (HeV M K258A) impairs FC-DFC localisation/Treacle-binding and DDR modulation/budding activity, without preventing localisation to the GC, where HeV M K258A accumulates [14]. K258 forms part of a bipartite nuclear localisation sequence (NLS; often referred to as ‘NLS2’; M contains at least two NLSs; NLS1 is located at residues 82–87 [16,17]) and is reported to be ubiquitinated [10,11,18]. This ubiquitination was recently shown to be mediated by the E3 ubiquitin ligase RAD18, in complex with the E2 ubiquitin-conjugating enzyme RAD6A [18]. Ubiquitination at K258 appears to also facilitate or influence the ubiquitination of the M protein at additional sites [10,11]. It has been proposed that the M protein enters the nucleus via the NLS and accumulates within nucleoli before exiting the nucleolus and nucleus (mediated by a nuclear export sequence (NES)). The exit of the nucleolus/nucleus and localisation at the plasma membrane is reported to be triggered by ubiquitination at K258 [18,19]. However, this model was proposed prior to the description of the functionally important localisation of HeV M to sub-nucleolar compartments. As a result, the coordination and regulation of various trafficking steps, including trafficking within the nucleolus, remain undefined.

The potential role of ubiquitination in sub-nucleolar localisation is of particular interest, as mechanisms regulating nucleolar/sub-nucleolar trafficking (which involves movement between LLPS structures) are poorly understood compared with those for nuclear trafficking, which involves conventional protein interactions with trafficking receptors and the nuclear pore complex. In this study, we examine the regulation of HeV M protein trafficking between the FC-DFC and GC, finding that ubiquitination plays a crucial role. Interestingly, our data indicate that ubiquitination exerts opposing effects on sub-nucleolar and nucleocytoplasmic localisation, suppressing exit from the FC-DFC to the GC while being required for egress from the nucleolus/nucleus.

## 2. Materials and Methods

### 2.1. Cell Culture, Transfection, and Treatment

HEK-293T (ATCC: CRL-3216) and HeLa (ATCC: CCL-2) cells were cultured in Dulbecco’s Modified Eagle Medium (DMEM) supplemented with 10% Fetal Calf Serum (FCS), 2 mM Glutamax, 50 U/mL Penicillin, and 50 μg/mL Streptomycin. The cells were maintained at 37 °C with 5% CO_2_. HEK-293T and HeLa cells were grown to 80–90% confluency before transfection using Lipofectamine 2000 and Lipofectamine 3000, respectively, according to the manufacturer’s instructions (ThermoFisher Scientific, Waltham, MA, USA). For free ubiquitin depletion, transfected HeLa cells were treated with 50 μM MG132 or 0.5% DMSO for control at 18 h p.t. for 6 h before CLSM imaging analysis. MG132 was purchased from Sigma (Sigma-Aldrich, St. Louis, MO, USA; M7449-200UL) as a 10 mM ready-made solution in DMSO. For experiments for the co-expression of HA-Ubi, HeLa cells were transfected with the same total amount of DNA of 2500 ng: 1000 ng of GFP-HeV M co-transfected with 1500 ng total of HA-alone plasmid and/or HA-Ubi, and the HA/HA-Ubi ratio varied.

### 2.2. Virus Infections

Wild-type HeV (Hendra virus/horse/1994/Hendra) was used for all virus work, which was performed at the CSIRO Australian Centre for Disease Preparedness (CSIRO-ACDP) in Biosafety Level (BSL)-4 laboratories. For the analysis of IF, HeLa cells were seeded onto coverslips and mock- or HeV-infected (MOI 5) prior to fixation at 7 h and 24 h p.i. using 4% paraformaldehyde (1 h, RT) and permeabilization with 0.1% TritonX-100 for 10 min. IF labelling was performed using a mouse primary antibody to HeV M (1:500; developed internally (Ref#: 1805-21-1527) and an anti-mouse AlexaFluor 488 secondary antibody. DNA was visualised using DAPI.

For the tissue culture infective dose (TCID_50_) analysis, HeLa cells were seeded into 96-well plates prior to HeV infection the next day at MOI 0.5 or 5. At 18 h, p.i. cells were treated with DMSO, MG132 (1 nM, 10 nM, or 100 nM), or Bortezomib (5 nM, 50 nM, or 500 nM). At 25 h p.i., an additional Bortezomib dose was added to Bortezomib samples, as previously performed for NiV [11]. At 42 h, p.i. supernatants were collected, and TCID_50_/mL was determined as previously described [20].

### 2.3. Constructs

The mammalian cell expression of N-terminal GFP tagged HeV-M (Accession Number AEB21196.1) and mutants were generated by the directional cloning of the M gene cDNA into the multiple cloning site of the pEGFP-C1 vector, as previously described [17]. The plasmid for expression of HA-ubiquitin (HA-Ubi) has been published previously [21].

### 2.4. Confocal Laser Scanning Microscopy (CLSM) and Image Analysis

For the CLSM imaging analysis, HeLa cells were seeded on 1.5 (0.17 mm) thickness glass coverslips and transfected with the indicated constructs at 80–90% confluency. Imaging was performed at the indicated time p.t. or 24 h p.t., if not specified. CLSM was conducted using a Nikon C1 inverted confocal microscope (Nikon Corporation, Tokyo, Japan) with a 60× oil immersion objective (NA 1.4) at Monash Micro Imaging Facility. Live-cell CLSM imaging was performed within a heated chamber at 37 °C.

CLSM images were analysed using ImageJ freeware software (version 2.1.0/1.53c). The mean fluorescence of the nucleus (Fn), cytoplasm (Fc), nucleolus (Fnu; whole nucleolus), FC-DFC (F_FC-DFC_), GC (F_GC_), and background fluorescence (Fb) were determined. After subtracting the background fluorescence (Fb) from all values, the nuclear to cytoplasmic (Fn/c), nucleolar to nuclear (Fnu/n), and FC-DFC to GC (F_FC-DFC/GC_) fluorescence ratios were calculated. In cells where accumulation into sub-nucleolar compartments was not evident (e.g., cells expressing K258A- or K258R-mutated M protein), two distinct areas in the diffuse region of the nucleolus were selected to represent the “FC-DFC” and the “GC” for image analysis. The F_GC_ analysis was based on images captured under the same microscopy and software settings.

The percentage of cells with FC-DFC accumulation was determined by dividing the number of cells showing any nucleolus with FC-DFC ≥ 1 by the total number of cells expressing the indicated proteins in each sample. Data are presented as mean ± S.E.M. (standard error of the mean) or mean ± SD (standard deviation), as indicated in the figure legend. Statistical analysis (Student’s *t*-test) was performed using GraphPad Prism software (version 10.4.1).

### 2.5. Immunofluorescence (IF)

For IF staining, cells grown on glass coverslips were washed twice gently with PBS at 24 h p.t., fixed with 4% (*w*/*v*) paraformaldehyde at room temperature (RT) for 15 min, permeabilized using 0.25% Triton X-100 (*v*/*v* in PBS) at RT for 5 min, and blocked with 1% bovine serum albumin (BSA) in PBS at RT for 1 h. For samples expressing FLAG-M proteins, an additional 5 min incubation with 5 μg/mL proteinase K was performed after fixation in order to visualise nucleolar localisation [22]. The cells were then incubated with a primary antibody specific to either Treacle (1:100; Cat # 11003-1-AP, Proteintech, Rosemont, IL, USA), UBF1 (1:500; Cat# Ab244287; Abcam, Cambridge, UK), Nucleolin (1:200; Cat#14574, CST, Thames Valley, UK), anti-FLAG (1:250; Cat#F1804, Sigma), or NPM1 (1:200; Cat# 32-5200, ThermoFisher Scientific) at RT for 1.5 h. Subsequently, cells were incubated with goat anti-rabbit or anti-mouse 568 AlexaFluor conjugate secondary antibody (Cat # A-11011/A-11004, ThermoFisher Scientific, Waltham, MA, USA) at a 1:1000 dilution in the dark at RT for 1.5 h. DNA staining was performed using Hoechst 33342 at a 1:2000 dilution of a 20 mM stock solution (Cat # 62249, ThermoFisher Scientific). The cells were mounted onto microscope glass slides (Lomb Scientific, Scoresby, VIC, Australia) using Mowiol reagent (Kuraray Europe GmbH, Hattersheim am Main, Germany).

### 2.6. 5-Ethnyl Uridine (EU) Incorporation Assays

Levels of rRNA synthesis were determined using an image-based technique (Click iT RNA Alexa Fluor 594 Imaging kit, Thermo-Fisher Scientific (Waltham, MA, USA), Cat# C10330), as previously described [14,23,24,25]. Cells were incubated for 1 h in the presence of EU before fixation in 4% paraformaldehyde at RT for 12 min and permeabilization in 0.25% Triton X-100 for 5 min at RT. Samples were processed according to the manufacturer’s recommendations to label incorporated EU with Alexa Fluor 594. DNA was labeled using Hoechst 33342. Cells were imaged by CLSM to detect the labeling of nascent rRNA by measuring the fluorescence intensity of Alexa Fluor 594 within nucleoli. The quantitative analysis was performed using ImageJ software (version 2.1.0/1.53c) to determine the mean EU fluorescence of nucleoli, which were identified using a combination of DNA, GFP, and transmitted light channels. Relative rRNA synthesis levels were determined by measuring nucleolar EU levels of both GFP-expressing and non-expressing cells from the same sample (as an internal control) and expressed as the nucleolar EU of GFP-expressing cells relative to non-expressing cells.

## 3. Results

### 3.1. Ubiquitination Affects Sub-Nucleolar Trafficking of HeV M Protein

Previously, the HeV M protein was reported to be ubiquitinated at several sites, potentially including residue K258 (and equivalent residues in other henipaviruses), and that the mutation of K258 to A or R (the latter preventing ubiquitination but retaining the positive charge) inhibits the ubiquitination of several sites that were shown to be mono-ubiquitinated [10,11]. Ubiquitination was implicated in M protein trafficking through the use of the proteasome and ubiquitination inhibitor MG132, which was shown biochemically to inhibit M ubiquitination, cause the nuclear and nucleolar retention of HeV and NiV M proteins, and block nuclear export and virus-like particle (VLP) production by NiV M [10,11].

To explore the possibility that ubiquitination regulates trafficking between sub-nucleolar condensates, we examined the effect of MG132 treatment on the localisation/accumulation of HeV M proteins to sub-nucleolar punctate compartments (which correspond to FC-DFC; Figure S1 and previously reported data [14]) using a confocal laser scanning microscopy (CLSM) analysis of living HeLa cells expressing GFP-fused wild-type (WT) HeV M (GFP-HeV M). GFP-HeV M accumulated with FC-DFC (Figure 1b). Notably, the FLAG-tagged HeV M protein also localised within the FC-DFC (Figure S2A) and interacted with Treacle (Figure S2B), but not with the Treacle-binding mutant (K258A), as expected [14]. These observations indicate that the GFP tag does not substantially alter M protein localisation or function, thereby validating the use of GFP-tagged M for live-cell imaging and other experiments throughout this study.

As expected [10,11], MG132 treatment resulted in an apparent increase in the nuclear accumulation of GFP-HeV M protein (Figure 1b); the quantitative image analysis confirmed a significant increase in the nuclear to cytoplasmic fluorescence ratio (Fn/c) (Figure 1c). This is consistent with the previously reported impairment of nuclear export [10,11,18]. In contrast, the accumulation of M proteins in the FC-DFC appeared to be reduced (Figure 1b), and this effect was confirmed by a reduced ratio of the fluorescence intensity of the FC-DFC compared with the GC (F_FC-DFC/GC_) (Figure 1d), an increase in GC fluorescence, indicative of the movement of M proteins from the FC-DFC into the surrounding GC (Figure 1e), and a decrease in the number of cells with the FC-DFC accumulation of M proteins (Figure 1f).

To confirm that the effects of MG132 on HeV M sub-nucleolar trafficking are due to ubiquitination, we co-transfected cells with a plasmid expressing HA-ubiquitin (HA-Ubi) to replenish ubiquitin depletion by MG132 (Figure 1g,h). Notably, MG132 more potently reduced F_FC-DFC/GC_ accumulation in this experiment (to ~1), compared to Figure 1d. This likely reflects the lower expression of HeV M due to the co-transfection of two plasmids, rendering cells more responsive to MG132 treatment. The expression of HA-Ubi reversed the effect of MG132 in reducing FC-DFC accumulation in a dose-dependent fashion (Figure 1g,h). These findings indicate that ubiquitination promotes the accumulation of the HeV M protein within the FC-DFC, while reduced ubiquitination leads to its egress from the FC-DFC and accumulation in the GC. The observed increase in GC fluorescence is consistent with previous reports of an apparent enhancement of the nuclear and nucleolar localisation of M proteins following MG132 treatment, which was proposed to reflect the decreased export from the nucleus and corresponding decrease in egress from the nucleolus [10]. Our data suggest that the accumulation of diffuse (GC) fluorescence in the nucleolus following MG132 treatment is not solely due to reduced nuclear/nucleolar egress, but also to increased egress from the FC-DFC to the GC. Notably, the opposing effects of ubiquitination on FC-DFC and nuclear/nucleolar GC localisation indicate different mechanisms affecting trafficking between the compartments, such that FC-DFC localisation is not simply the result of altered protein concentration in the nucleus/nucleolar compartment but is specifically and distinctly regulated by ubiquitination.

Previously, proteosome inhibitors were shown to reduce NiV titers during live virus infections, indicating that ubiquitination plays a critical role in NiV infection [11]. To test if similar mechanisms occur during HeV infection, HeLa cells were infected with HeV at MOIs of 0.5 or 5, followed by treatment with proteosome inhibitors MG132 (Figure 1i) and Bortezomib (Figure 1j). Both inhibitors reduced virus titers in a dose-dependent manner at both MOIs, with statistically significant effects at both MOIs for Bortezomib and at MOI 0.5; the reduction observed for MG132 at MOI 5 was dose-dependent but not significant. These findings confirm the importance of ubiquitination in HeV infection, similar to what is observed with NiV [11].

### 3.2. Conservative Substitution of K258 to R Reduces FC-DFC Targeting by HeV M

**Figure 2:**
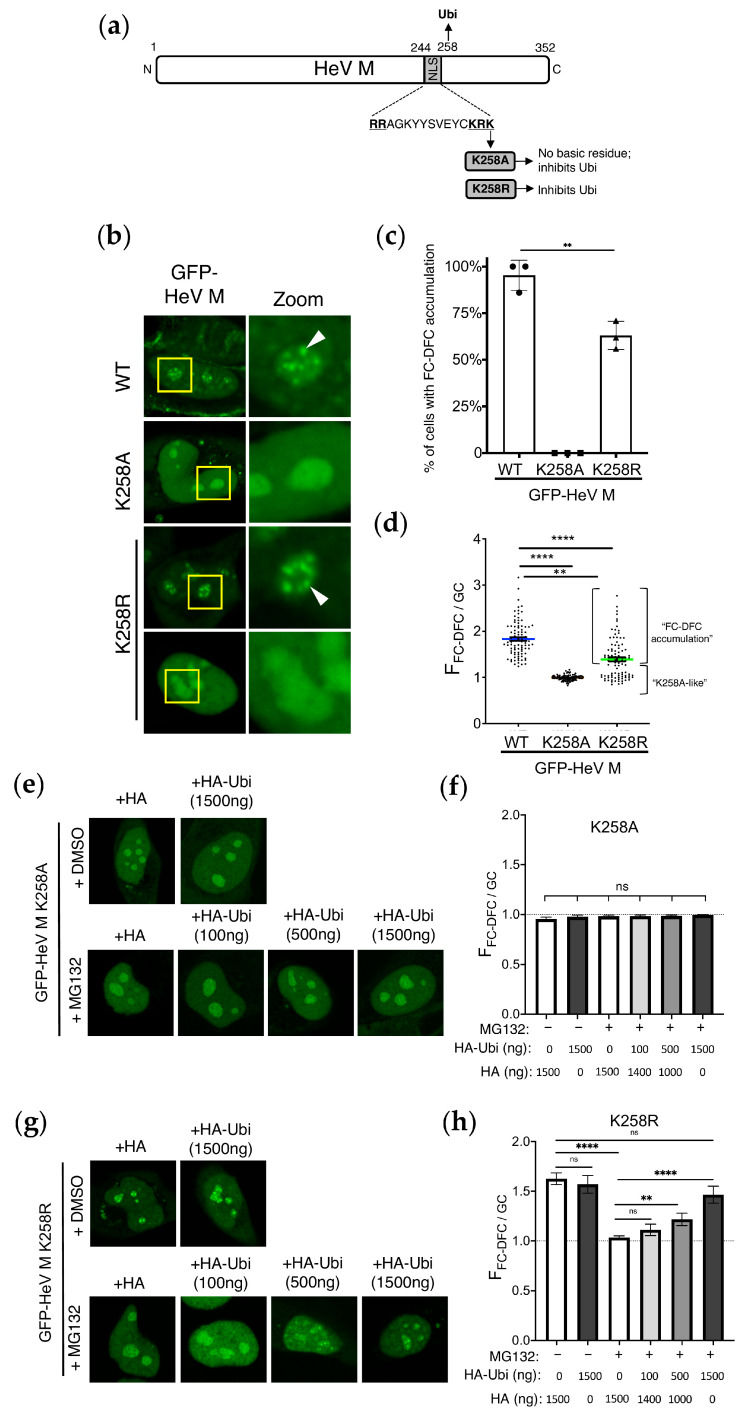
**K258R mutation impacts sub-nucleolar trafficking of HeV M protein**. (**a**) Schematic of the HeV M protein showing the bipartite NLS (residues 244–258; critical basic residues are bolded and underlined), and residue K258, which undergoes ubiquitination (Ubi). Substitution of K258 (e.g., to A or R) removes the ubiquitination site. Mutations used in this study are indicated (grey boxes). (**b**) CLSM images of living HeLa cells expressing the indicated GFP-fused proteins (24 h p.t.); for HeV M WT and K258A, images are representative of 90–100% of cells in > 29 fields of view; for HeV M K258R, two major populations (each representing c. 40–60% of the population) were observed, corresponding to either a “FC-DFC accumulation” (upper panel) or “K258A-like” (lower panel) phenotype. Nucleoli are highlighted by the yellow box, which is magnified in the zoom panel. White arrowheads indicate accumulation within FC-DFC. (**c**) Images such as those in B were analysed to determine the percentage of cells with clear FC-DFC accumulation of M protein (mean percentage ± SD, *n* = 3 separate assays, each sampling ≥ 119 cells). Student’s *t*-test with Welch’s correction was used to determine significance; ** *p* < 0.01). (**d**) Images were analysed to determine F_FC-DFC/GC_ (mean ± S.E.M., *n* ≥ 90 cells for each condition, from three independent assays; green line in K258R indicates mean of all samples). The two distinct populations in the K258R sample are indicated. Comparison of K258R “WT-like” population with WT samples using Student’s *t*-test showed a significant difference (*p* < 0.01). (**e**–**h**) Images of HeLa cells co-transfected to express GFP-HeV M K258A (**e**) or K258R (**g**) with different amounts of HA or HA-ubiquitin-expressing plasmid imaged by CLSM (as in Figure 1g,h). Images such as these were analysed to determine the F_FC-DFC/GC_ of K258A (**f**) and K258R (**h**) expressing cells (*n* ≥ 17 cells for F, and *n* ≥ 21 for H; data from one assay, representative of two independent assays). Dashed line indicates F_FC-DFC/GC_ of 1. Statistical analysis used Student’s *t*-test; ** *p* < 0.01; **** *p* < 0.0001; ns, non-significant.

We previously showed that K258A mutation in the HeV M protein (HeV M K258A) abolishes its targeting to the FC-DFC, resulting in accumulation within the GC and loss of binding to the FC-DFC-enriched protein, Treacle [14]. This suggested that K258 forms part of a targeting signal due to its positive charge and/or affects sub-nucleolar localisation due to its ubiquitination [10,11]. The above data (Figure 1) indicate that ubiquitination is required for the retention of HeV M within the FC-DFC. Mutation at K258 is reported to affect mono-ubiquitination at K258 and several other sites, suggesting that K258 also impacts on other mono-ubiquitination sites in M proteins [11,18]. To examine whether the effects we observed on FC-DFC localisation following MG132 relate to ubiquitination at K258 or associated sites, we compared the effects on the subcellular localisation of HeV M by the substitutions K258A (which removes the positive charge and the potential ubiquitination site) and K258R (which retains a positive charge but lacks the lysine of the potential ubiquitination site) (Figure 2a). Previous studies on equivalent mutations in the NiV M protein indicated that the positive charge is important to the function of the nuclear localisation sequence (NLS) and nucleolar accumulation, while ubiquitination regulates nuclear export [11]. However, no effects on sub-nucleolar localisation were reported, although our data indicate that ubiquitination has opposing effects on nuclear/nucleolar accumulation and FC-DFC accumulation (above).

The CLSM analysis of cells expressing GFP-fused HeV M WT, K258A, or K258R variants (Figure 2b), indicated the sub-nucleolar accumulation of WT M proteins in c. 90% of cells, consistent with localisation to FC-DFC (Figure 2c). The Western blot analysis confirmed that the expression levels of GFP-fused WT and mutant proteins were broadly comparable under these conditions (Figure S3), indicating that differences in localisation are not attributable to variation in protein expression. As expected [14], HeV M K258A proteins did not localise/accumulate within FC-DFC, but accumulated within the GC in 100% of cells (Figure 2c). In contrast, HeV M K258R displayed an intermediate phenotype, with a substantial proportion of cells (c. 60%) showing FC-DFC accumulation, similar to WT, and the remainder lacking FC-DFC accumulation, similar to K258A (Figure 2b,c). Consistent with this, the F_FC-DFC/GC_ ratio for HeV M K258A (c. 1.0) was significantly lower than that for HeV M WT (c. 1.8), while HeV M K258R showed an intermediate phenotype (c. 1.4) (Figure 2d). The reduced F_FC-DFC/GC_ for HeV M K258R resulted from the presence of a K258A-like sub-population (for which the F_FC-DFC/GC_ was equivalent to that for K258A) and the fact that the F_FC-DFC/GC_ for the population with apparent FC-DFC accumulation was significantly lower than the F_FC-DFC/GC_ for HeV M WT proteins (*p* < 0.01) (Figure 2d); thus, even in cells where HeV M K258R localised to the FC-DFC, this localisation was impaired compared with the HeV M WT protein. Notably, this intermediate localisation of HeV M K258R was paralleled by its functional activity. Specifically, HeV M K258R inhibited rRNA biogenesis to an extent intermediate between HeV M WT (~25% inhibition) and K258A (no inhibition) (Figure S4A,B), supporting a correlation between FC-DFC localisation and functional output.

The overexpression of HA-Ubi, treatment with MG132, or a combination of these conditions did not result in any significant FC-DFC accumulation of HeV M K258A (Figure 2e,f), consistent with the positive charge at residues 258 being essential for FC-DFC localisation [14]. Interestingly, the MG132 treatment of cells expressing GFP-HeV-M-K258R resulted in a significant reduction in FC-DFC accumulation to reach levels similar to HeV M K258A and HeV M WT with MG132/HA (Figure 1h). The expression of HA-Ubi reversed this effect (Figure 2g,h). Together, these data imply that ubiquitination dependent on K258 is required for efficient FC-DFC localisation, and further suggest that ubiquitination at other K258-independent sites (either within M protein or in other cellular proteins) may also contribute to this process.

### 3.3. Dynamic Localisation of HeV M in Sub-Nucleolar Compartments Is Regulated by K258

**Figure 3:**
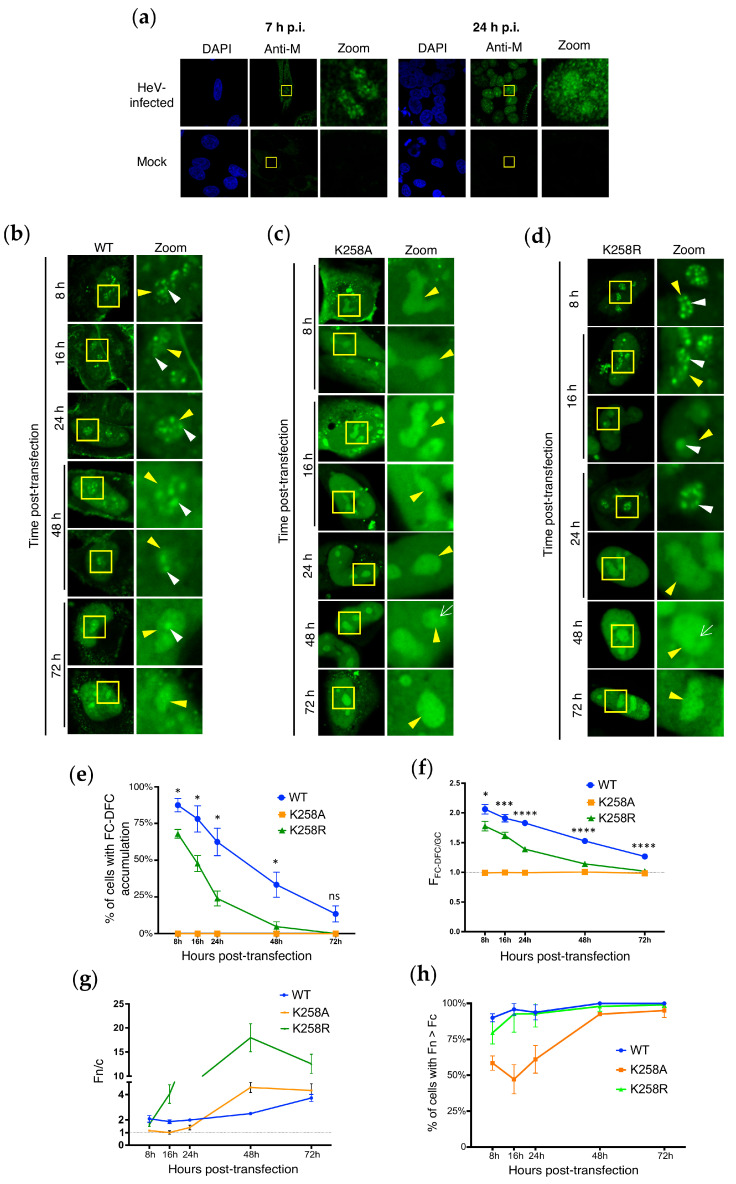
**HeV M protein undergoes dynamic localisation to the FC-DFC, which is impacted by K258R mutation.** (**a**) HeLa cells were mock-infected or infected with HeV (MOI 5) prior to fixation and immunostaining for HeV M protein at 7 h and 24 h post-infection (p.i.). Nuclei were detected using DAPI (blue). Microscope settings and image correction are identical between equivalent mock and HeV-infected images. (**b**–**d**) HeLa cells transfected to express the indicated proteins were analysed live at 8, 16, 24, 48, and 72 h p.t. by CLSM. Images representative of major phenotypes are shown for each condition; yellow boxes are magnified in the zoom panel. Yellow arrowheads indicate nucleoli; white arrowheads indicate accumulation of M protein in FC-DFC; white arrow indicates absence of M in FC-DFC. Images such as those in (**b**–**d**) were analysed to determine the following: (**e**) the percentage of M protein-expressing cells with FC-DFC accumulation in any nucleolus in the cell (mean ± SD from three independent assays, *n* ≥ 59 cells for each condition); (**f**) F_FC-DFC/GC_ (mean ± S.E.M., *n* ≥ 55 cells for each condition from three independent assays, except for 8 h p.t. (WT, K258A, K258R), 16 h p.t. K258A, and 48 h p.t. WT samples, where data are from two assays; (**g**) Fn/c (mean ± S.E.M.; *n* ≥ 24 cells); and (**h**) percentage of cells with Fn > Fc (mean ± SD from three independent assays, except for 8 h p.t. (WT, K258A, K258R), 16 h p.t. K258A, and 48 h p.t. WT samples, where data are from two assays; *n* ≥ 23 cells for each condition). Student’s *t*-test was used to compare values for WT and K258R at each time point in (**e**,**f**); * *p* < 0.05; *** *p* < 0.001; **** *p* < 0.0001; ns, non-significant.

The effects of the K258 mutation on HeV M protein accumulation in FC-DFC suggest potential impacts on a targeting sequence and/or affinity for specific components within the FC-DFC. For NiV M proteins, K258 is proposed to be part of a NLS, which typically consists of short stretches of basic residues. Several basic residues (R244, R245, R256, R257, K258 in NiV M) are highly conserved among henipavirus M proteins [10,11]. Thus, K258 may play a crucial role in nuclear import through the NLS and contribute to an overlapping targeting sequence for nucleoli/sub-nucleolar FC-DFC [11,17]. Previous data indicated that ubiquitination dynamically regulates the nuclear localisation of HeV M [10]. Moreover, the NiV M protein undergoes the dynamic and temporal regulation of localisation during infection, being nuclear/nucleolar early in infection before nucleolar exit/nuclear export, and eventual accumulation and budding at the plasma membrane [9,11]. Similarly, we found that in HeV-infected cells, HeV M localises to sub-nucleolar compartments (FC-DFC) early during infection (7 h post-infection (p.i.)), but becomes more diffuse in the nucleolus (i.e. accumulated into the GC), and with greater nuclear accumulation by 24 h p.i. (Figure 3a), consistent with observations for GFP HeV M WT protein and observations of dynamic nuclear/nucleolar localisation of M protein in NiV-infected cells [11]. Thus, we speculated that the observed differences in FC-DFC localisation between HeV M WT and the K258 mutants (assessed at 24 h post-transfection in Figure 1 and Figure 2) might be attributable to the dynamic regulation of various M protein trafficking signals related to the changes in HeV M localisation during infection.

To investigate this, we assessed the sub-nucleolar localisation of HeV M WT and mutant proteins at time points from 8 h to 72 h p.t. (Figure 3b–d). The WT HeV M protein exhibited clear accumulation in FC-DFC (typically multiple structures in each nucleolus) at 8 h p.t. (c. 85% of cells), which progressively diminished over the course of the experiment, accompanied by a more diffuse GC distribution with only around 10% of cells exhibiting the accumulation of M proteins in multiple FC-DFCs at 72 h p.t. (Figure 3e). This is consistent with a dynamic interaction whereby M protein initially enters the FC-DFC and then undergoes gradual egress to the GC. The measurement of the F_FC-DFC/GC_ confirmed a progressive loss of FC-DFC localisation (Figure 3f). This correlated with the known functional implications of HeV M localisation to the FC–DFC in inhibiting rRNA biogenesis [14]; inhibition observed at 24 h p.t. was no longer evident by 72 h p.t. (Figure S4C).

Consistent with roles of K258 in NLS activity of NiV M protein [11], the Fn/c for HeV M K258A was reduced compared with WT at 8 and 16 h p.t., supporting its involvement in nuclear import (Figure 3g; Fn/c c. 2 for WT, compared with Fn/c c. 1 for K258A at both time points). Further analysis revealed that the reduction in the nuclear localisation of the K258A mutant was due to a significant proportion of cells with higher fluorescence intensity in the cytoplasm (Fc) than in the nucleus (Fn) at early time points, in contrast to cells expressing WT and K258R M proteins (Figure 3h; c. 50–60% of cells expressing K258A M protein showed Fn > Fc between 8 and 24 h p.t., whereas in cells expressing WT M protein, >85% of cells showed Fn > Fc at all time points). However, over time, the K258A mutant gradually exhibited a proportion of cells with Fn > Fc similar to WT and K258R (nearly 100% of cells at 48 and 72 h p.t.), suggesting a delay in the nuclear import of K258A compared to the other variants.

Despite reduced nuclear accumulation at early time points, HeV M K258A was strongly nucleolar at all time points, consistent with reduced nucleolar egress. However, no accumulation in FC-DFC was observed at any time point, and some images indicated the absence of fluorescence from these structures (e.g., white arrow, 48 h p.t., Figure 3c). Thus, HeV M proteins can specifically partition between sub-nucleolar phase-separated compartments, dependent on K258, and this is independent of the accumulation in the nucleus, consistent with distinct mechanisms of trafficking/localisation. **To determine whether this property is conserved and not HeV-specific, we also examined NiV M, which showed similar dynamics**—notably, the percentage of cells showing FC-DFC accumulation reducing over time (c. 25% of cells showing FC-DFC accumulation at 72 h p.t.), while NiV M-K258A remained excluded from FC-DFC at all time points (Figure S5).

HeV M K258R accumulated to higher levels in the nucleus than the cytoplasm at early time points compared with K258A (Figure 3d, upper panels and 3G), similar to WT M. This is consistent with a requirement for the positive charge in the NLS for efficient nuclear import, as reported for NiV M proteins [11,17]. Additionally, HeV M K258R accumulated to very high levels in the nucleus at later time points (48 h), consistent with an impaired nuclear export mechanism [10,11]. HeV M K258R also showed clear FC-DFC localisation in Treacle-enriched compartments at 8 h p.t. (Figure 3d), similar to (but moderately reduced compared with) WT HeV M proteins, followed by the loss of FC-DFC localisation over time. Thus, the presence of a basic residue at position 258 is necessary for initial entry and accumulation within the FC-DFC.

While FC-DFC localisation of WT and K258R HeV M proteins diminished following the initial accumulation, the apparent rate of loss was greater for HeV M K258R, such that by 24 h p.t. (Figure 3e), c. 25% of HeV M K258R-expressing cells displayed FC-DFC localisation compared with c. 60% for WT HeV M. By 48 and 72 h p.t., <5% and 0%, respectively, of HeV M K258R-expressing cells displayed FC-DFC localisation, and nucleoli with apparent exclusion from FC-DFC structures were apparent (e.g., 48 h p.t., Figure 3d, white arrow, similar to observations for HeV M K258A). The calculation of the F_FC-DFC/GC_ ratio confirmed a significant decrease in FC-DFC localisation by both HeV M WT and K258R over the course of the experiment, with a more rapid decrease for the latter (Figure 3f). Thus, it appears that HeV M localises initially to the FC-DFC, dependent primarily on the presence of a positive charge at position 258. HeV M then relocalises to the GC, and this process is accelerated in HeV M containing the K258R substitution that is impaired for ubiquitination, consistent with ubiquitination supporting retention into the FC-DFC. This model also accounts for the two distinct populations observed for K258R (Figure 2d), which likely reflect cells at different stages of this trafficking pathway.

### 3.4. Loss of HeV M FC-DFC Accumulation Does Not Relate to Disruption or Loss of FC-DFC

**Figure 4:**
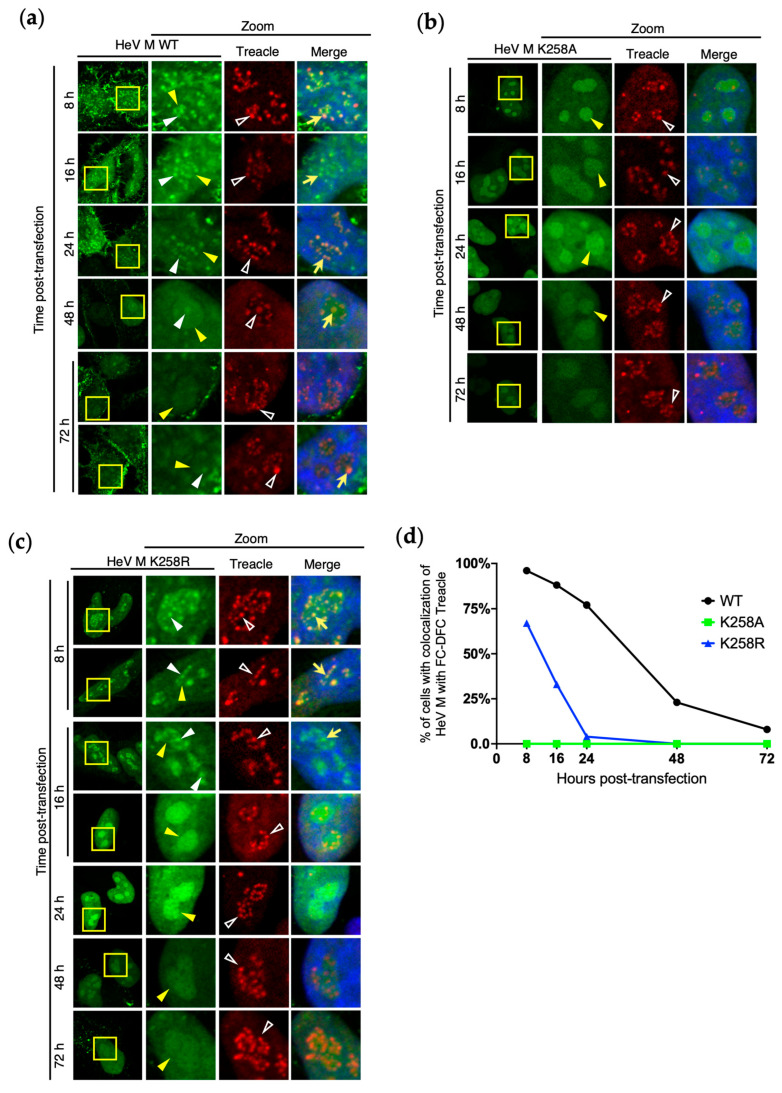
**M protein FC-DFC accumulation decreases over time without loss of FC-DFC compartments.** (**a**–**d**) HeLa cells were transfected to express the indicated proteins before fixation at 8, 16, 24, 48, and 72 h p.t. and immunostaining using anti-Treacle antibody (red) and imaging via CLSM. Hoechst 33342 (blue in Merge panels) was used to localise DNA/nuclei. Representative images are shown for each condition; yellow arrowheads indicate nucleoli; unfilled white arrowheads indicate Treacle in FC-DFC; white arrowheads indicate accumulation of M protein into FC-DFC; yellow arrows indicate colocalisation of Treacle and HeV M protein in FC-DFC. Images such as those in (**a**–**d**) were analysed to determine the percentage of cells expressing HeV M protein with evident colocalisation of HeV M protein and Treacle in FC-DFC (*n* ≥ 23 cells for each condition).

The loss of HeV M FC-DFC localisation (Figure 3) could be attributed to two possible mechanisms: (1) the egress of the protein from intact FC-DFC structures, or (2) the depletion of FC-DFC structures through events such as the fusion or disassembly/disruption of the liquid bodies. To investigate these possibilities, we analysed cellular FC-DFCs directly by the fixation and immunostaining of cells for Treacle at time points from 8–72 h p.t. to express HeV M WT, K258A, and K258R (Figure 4a–c).

In cells expressing GFP-HeV M WT proteins, the appearance of FC-DFCs was similar throughout the experiment (Figure 4a). At early time points, HeV M WT proteins strongly colocalised with Treacle in FC-DFC, but this diminished over time (indicated by a reduced percentage of cells with detectable colocalisation of HeV M and Treacle FC-DFCs), although multiple Treacle-enriched FC-DFCs lacking HeV M association remained detectable in nucleoli (Figure 4a,d). Thus, the HeV M protein appears to transit through FC-DFC, where it has previously been shown to interact with Treacle [14], before egress, with no significant disruption of FC-DFC structures. As expected, HeV M K258A showed no colocalisation/accumulation in Treacle FC-DFC at any time point, despite the presence of multiple FC-DFCs. HeV M K258R proteins showed similar results to WT, but with more rapid egress, as expected (Figure 3c), and no evident loss of FC-DFC (Figure 4c,d). By 24–48 h p.t., colocalisation was barely detectable, similar to K258A (Figure 4d). Thus, it appears that the HeV M protein transits through intact FC-DFC, with the loss of colocalisation due to trafficking rather than disruption or major structural change to FC-DFC. These data further support that the dynamic localisation of M protein to FC-DFC, and interaction with Treacle, underlie the specific silencing of rRNA biogenesis.

### 3.5. Ubiquitination Regulates Sub-Nucleolar Trafficking of M Proteins of Multiple Henipaviruses

**Figure 5:**
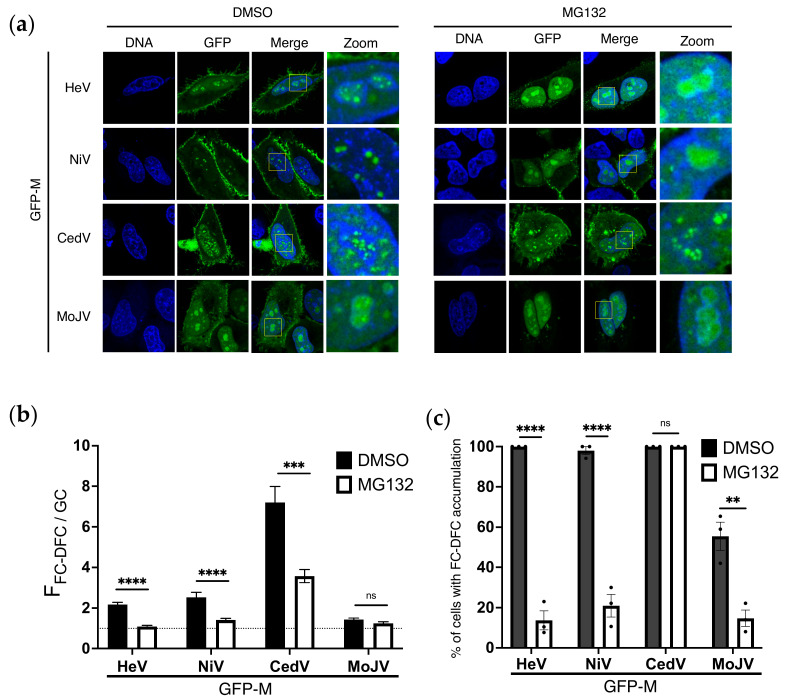
**FC-DFC accumulation of M proteins of multiple henipaviruses is regulated by ubiquitination.** (**a**) CLSM images of live HeLa cells transfected to express the indicated proteins. Representative images are shown for each condition, with yellow boxes magnified in the zoom panel. Hoechst 33342 was used to stain nuclei/DNA (blue). (**b**) Images such as those in A were used to determine F_FC-DFC/GC_ (mean F_FC-DFC/GC_ ± S.E.M., from one assay (*n* ≥ 13), representative of three independent assays). Dashed line indicates F_FC-DFC/GC_ of 1. (**c**) The percentage of M protein-expressing cells with apparent accumulation of M protein in FC-DFC (mean percentage ± S.E.M. from three independent assays; each sample was determined from *n* ≥ 13 cells). ** *p* < 0.01; *** *p* < 0.001; **** *p* < 0.0001; ns, non-significant.

Previously, we showed that the FC-DFC accumulation, Treacle binding, and inhibition of rRNA biogenesis are conserved among M proteins of multiple henipaviruses (including NiV, Cedar (CedV), and Mojiang (MojV) viruses), albeit with some differences in the extent of FC-DFC accumulation [25]. To determine if the ubiquitin-dependence of FC-DFC accumulation is conserved in different henipaviruses, we assessed the effects of MG132 as above (e.g., Figure 1). Similar to HeV M, MG132 treatment significantly impaired NiV M FC-DFC accumulation (Figure 5a,b) and reduced the percentage of cells with FC-DFC accumulation (Figure 5c), consistent with the homology of HeV and NiV M proteins (~90% amino acid identity). Consistent with our previous report [25], CedV M showed the highest accumulation in FC-DFC and lowest GC accumulation of the M proteins assessed; the F_FC-DFC/GC_ accumulation of CedV M proteins was significantly reduced (but remained higher than that of HeV or NiV M proteins) following MG132 treatment, and the percentage of cells with FC-DFC accumulation of CedV M proteins remained c. 100% (Figure 5c). MojV M showed the lowest accumulation in FC-DFC (consistent with previous data [25]), resulting in only a minor and non-significant reduction in F_FC-DFC/GC_ (Figure 5b); however, there was a significant reduction in the percentage of cells, with the clear FC-DFC accumulation of MojV M proteins following MG132 treatment (Figure 5c). Taken together, these data indicate the conserved roles of ubiquitination in regulating henipavirus M protein localisation to the FC-DFC, although the extent of accumulation differs between M proteins, correlating with evolutionary divergence (c. 61% and 60% similarity of CedV and MojV M proteins, respectively, compared with HeV M protein).

## 4. Discussion

Here, we have found that the HeV M protein dynamically transits through the FC-DFC, indicating that the previously identified translocation through the nucleus/nucleolus involves additional sub-nucleolar trafficking between LLPS structures, with the different stages of trafficking regulated by post-translational modification. This transit enables regulated interactions with Treacle and other host factors, enabling the functional regulation of rRNA synthesis by modulating the nucleolar DDR, as well as virus assembly and budding. To our knowledge, this study presents the first data on the mechanisms governing the trafficking of a viral protein within sub-nucleolar LLPS structures and expands and refines the model for M protein trafficking. Specifically, we found that M trafficking to FC-DFC requires a basic residue at residue 258, and that its egress from FC-DFC is regulated, at least in part, by the ubiquitination status of the M protein. Importantly, while ubiquitination has previously been reported to regulate nuclear and overall nucleolar localisation [10], indicating that ubiquitination is required for efficient nuclear export and nucleolar egress, our data, in contrast, indicate that ubiquitination is required for nucleolar retention/accumulation into the FC-DFC. These findings highlight that the localisation of proteins to specific sub-nucleolar compartments involves highly specific mechanisms.

Taken together, our data support a model for M protein sub-nucleolar trafficking in which the entry of M proteins into the FC-DFC and functional interaction with Treacle to modulate rRNA biogenesis requires a basic charge at residue 258. However, the retention/egress of M from the FC-DFC is dynamically regulated, at least in part, by the ubiquitination status of M proteins. Our data support a likely role for the ubiquitination of K258, but also indicate that other ubiquitination sites, either within M proteins or on host proteins, also contribute, as FC-DFC localisation by HeV M K258R proteins is impaired but remains sensitive to the inhibition of ubiquitination. These observations are consistent with previous data indicating that M proteins can be mono-ubiquitinated at at least four sites, and K258R mutation inhibits ubiquitination at several of these sites (proposed to include K258 itself), which is likely to contribute to the impaired FC-DFC localisation of this mutant. However, at least one site remains functional, which may account for the residual accumulation of HeV M K258R into FC-DFC that is lost following MG132 treatment [10,11]. Our data are consistent with a model whereby the M protein exits the FC-DFC but egress is negatively regulated by the ubiquitination of the M protein; however, it is also possible that the ubiquitination of host proteins also regulates interactions with M proteins required for exit.

The movement of proteins between LLPS MLO structures such as nucleolar sub-compartments does not use conventional translocation processes associated with membrane-enclosed organelles (e.g., movement via pores/channels), but rather depends on partitioning through physicochemical properties and interactions with MLO-resident molecules [4,26]. This likely accounts for the poor definition of nucleolar ‘targeting sequences’ compared with NLS and NES motifs that form specific interactions with trafficking receptor proteins (importins and exportins) [27]. Ubiquitination plays important roles in the formation and regulation of LLPS [28,29]; thus, our data could indicate ubiquitination may coordinate the M protein’s interactions/localisation into sub-nucleolar liquid bodies. Thus, ubiquitination may alter the physicochemical properties of M proteins or interactions with constituents of different nucleolar condensates, as well as affect importin/exportin interactions and interactions at budding sites. The differing nature of the mechanisms of sub-nucleolar trafficking and nucleocytoplasmic trafficking are consistent with our observations that ubiquitination has differing effects on the exit from the FC-DFC to the GC, nucleolus to the nucleus, and nucleus to the cytoplasm. Thus, the specific orchestration of ubiquitination, deubiquitination, and LLPS interactions may underlie the appropriate temporal regulation of transport between these compartments, enabling the specific control of rRNA silencing, virus replication, and assembly/budding, aligning with different stages of the viral life cycle [13].

As viruses typically mimic or hijack cellular processes, our findings likely have implications beyond viral infection. The intricate regulation of M proteins in multiple intra-nuclear compartments is unlikely to have evolved solely to control the concentration in the cytoplasm for viral processes such as assembly and budding. Rather, it suggests a specific coordination of sub-nuclear functions, including DDR subversion, where the M protein appears to mimic cellular NBS1 [14]. Our findings on the regulation of the sub-nucleolar partitioning of M protein, including roles of positively charged residues (typical of nuclear/nucleolar targeting signals) and ubiquitination, identifies mechanisms that may be relevant to cellular proteins that transit through sub-nucleolar compartments, including those of the DDR. In the light of current advances toward the therapeutic modulation of cellular and viral LLPS structures [30,31,32,33], our data also have the potential to contribute to novel antiviral approaches for currently incurable henipavirus infections, and possibly other nucleolus-related pathologies [34,35].

## Data Availability

The raw data supporting the conclusions of this article will be available on request.

## Supplementary Materials

The following supporting information can be downloaded at: https://www.mdpi.com/article/10.3390/v17060797/s1, Figure S1: GFP-HeV M protein accumulation colocalises with FC-DFC marker UBF1 but not GC markers; Figure S2: FLAG-HeV M binds Treacle and localises to sub-nucleolar compartments; Figure S3: Western blot analysis of GFP-M and mutants; Figure S4: Inhibition of rRNA biogenesis by HeV M protein correlates with dynamic localisation to the FC-DFC; Figure S5: FC-DFC accumulation of NiV M protein decreases over time.

## Abbreviations

The following abbreviations are used in this manuscript:

BSA	Bovine Serum Albumin
BSL	Biosafety Level
CedV	Cedar Virus
CLSM	Confocal Laser Scanning Microscopy
DFC	Dense Fibrillar Component
DMEM	Dulbecco’s Modified Eagle Medium
DMSO	Dimethyl Sulfoxide
DDR	DNA Damage Response
FC	Fibrillar Center
FC-DFC	Fibrillar Center–Dense Fibrillar Component
FCS	Fetal Calf Serum
Fn	Nuclear Fluorescence
Fc	Cytoplasmic Fluorescence
F_FC-DFC_	Fluorescence intensity in FC–DFC
F_GC_	Fluorescence intensity in Granular Component
GC	Granular Component
GFP	Green Fluorescent Protein
HA	Hemagglutinin
HeV	Hendra Virus
IF	Immunofluorescence
LLPS	Liquid–Liquid Phase Separation
MLO	Membrane-Less Organelle
MOI	Multiplicity of Infection
NLS	Nuclear Localisation Signal
NES	Nuclear Export Signal
NiV	Nipah Virus
p.i.	Post-Infection
p.t.	Post-Transfection
SD	Standard Deviation
SEM	Standard Error of the Mean
TCID_50_	Tissue Culture Infective Dose 50%
UBF1	Upstream Binding Factor 1
VLP	Virus-Like Particle
WT	Wild Type
