# Supplementary material for: Sub-Nucleolar Trafficking of Hendra Virus Matrix Protein Is Regulated by Ubiquitination
Source: Viruses. 2025 May 30;17(6):797. doi: 10.3390/v17060797 (PMC12197414; doi:10.3390/v17060797)
Supplement: Supplementary file 1 [file viruses-17-00797-s001.zip › viruses-3593090-supplementary.pdf]

Supplementary Materials for:

Sub-Nucleolar Trafficking of Hendra Virus Matrix Protein Is Regulated by Ubiquitination

Tianyue Zhao [1,†], Florian A. Gomez [1,†], Cassandra T. David [1], Christina L. Rootes [2], Cameron R. Stewart [2], Gregory W. Moseley [1,*,‡] and Stephen M. Rawlinson [1,*,‡]

1 Department of Microbiology, Biomedicine Discovery Institute, Monash University, 19 Innovation Walk, Clayton Campus, Clayton, VIC 3800, Australia;
2 Commonwealth Scientific and Industrial Research Organisation (CSIRO), Health and Biosecurity, Australian Centre for Disease Preparedness, Geelong, VIC 3219, Australia;
* Correspondence: greg.moseley@monash.edu (G.W.M.); stephen.rawlinson@monash.edu (S.M.R.)
† T.Z. and F.A.G. These authors contributed equally to this work.
‡ G.W.M. and S.M.R. These authors contributed equally to this work.

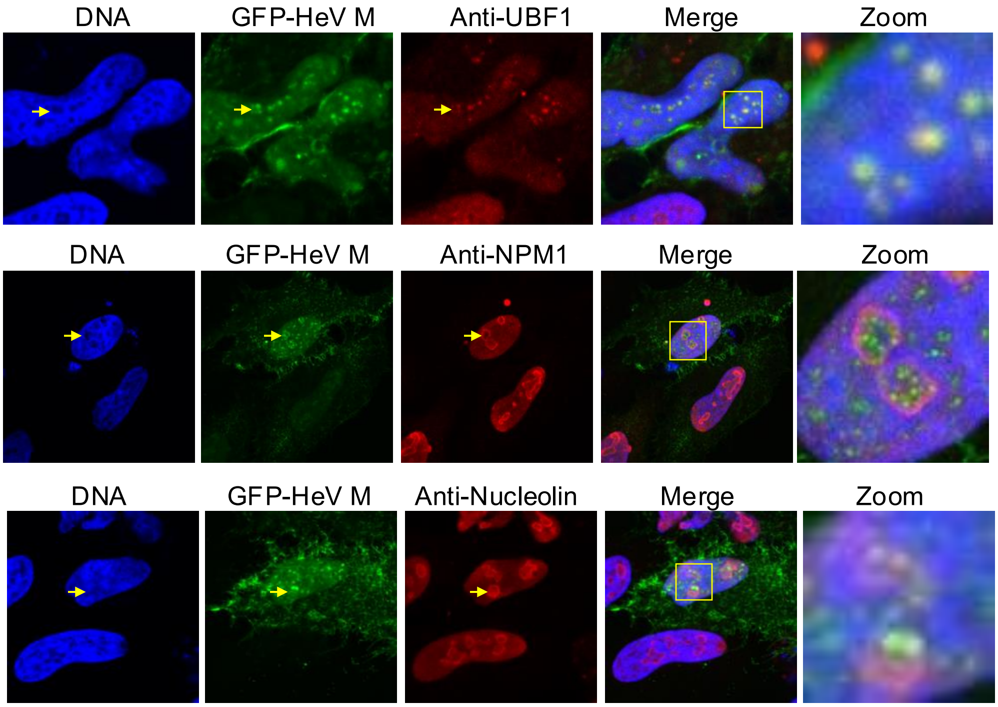

Figure S1. GFP-HeV M protein accumulation colocalizes with FC-DFC marker UBF1 but not GC markers.
HeLa cells were transfected to express GFP-HeV M and fixed at 24 h p.t. with 4% paraformaldehyde before immunostaining for the nucleolar markers UBF1 (FC-DFC localization) and NPM1 and nucleolin (GC localization). Yellow arrows highlighted selected nucleoli. Yellow boxes are magnified in the zoom panel. Hoechst 33342 (blue) was used to identify DNA/nuclei.

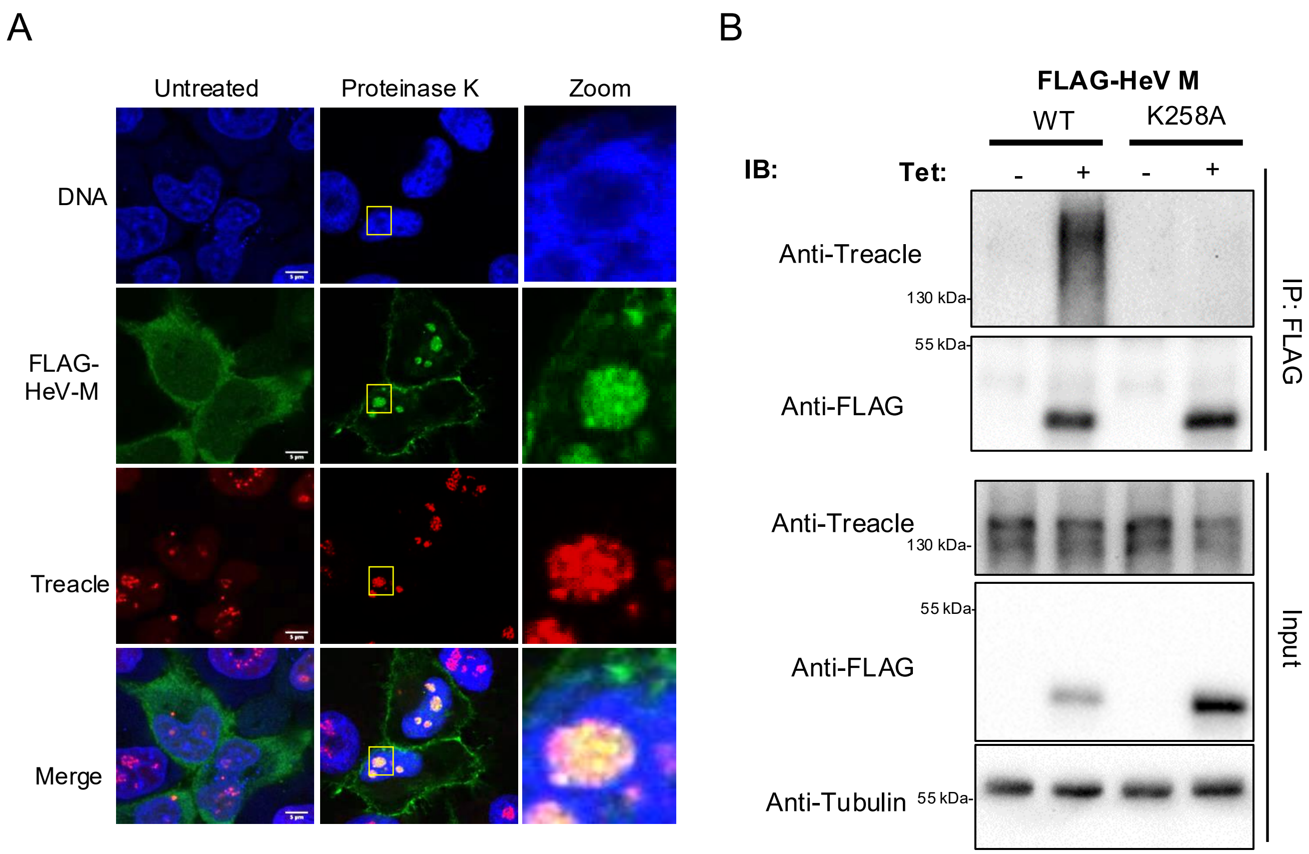

Figure S2. FLAG-HeV M binds Treacle and localizes to sub-nucleolar compartments. (A) HeLa cells transfected to express FLAG-HeV M were fixed with 4% paraformaldehyde and treated with or without proteinase K, before immunostaining for FLAG (green) and Treacle (red). Yellow boxes are shown magnified in the zoom panel. Hoechst 33342 (blue) was used to identify DNA/nuclei. (B) HEK293 FLP-In™ cells stably transfected to enable inducible expression of 3xFLAG-HeV M or 3xFLAG-HeV M-K258A were treated with tetracycline (+Tet) to induce expressions, or not treated (-Tet), for 24 h before lysis and IP for FLAG; IPs were analysed by IB using the indicated antibodies.

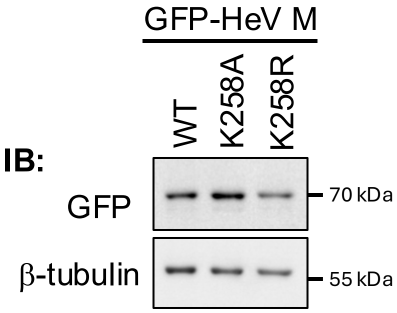

<u>**Figure S3**</u> **Western blot analysis of GFP-M and mutants**. HeLa cells were transfected to express the indicated GFP-tagged HeV M proteins. At 24 h post-transfection, cells were lysed in 2× SDS-PAGE sample buffer, and lysates were subjected to Western blotting using antibodies against GFP and β-tubulin. Molecular weight markers are indicated.

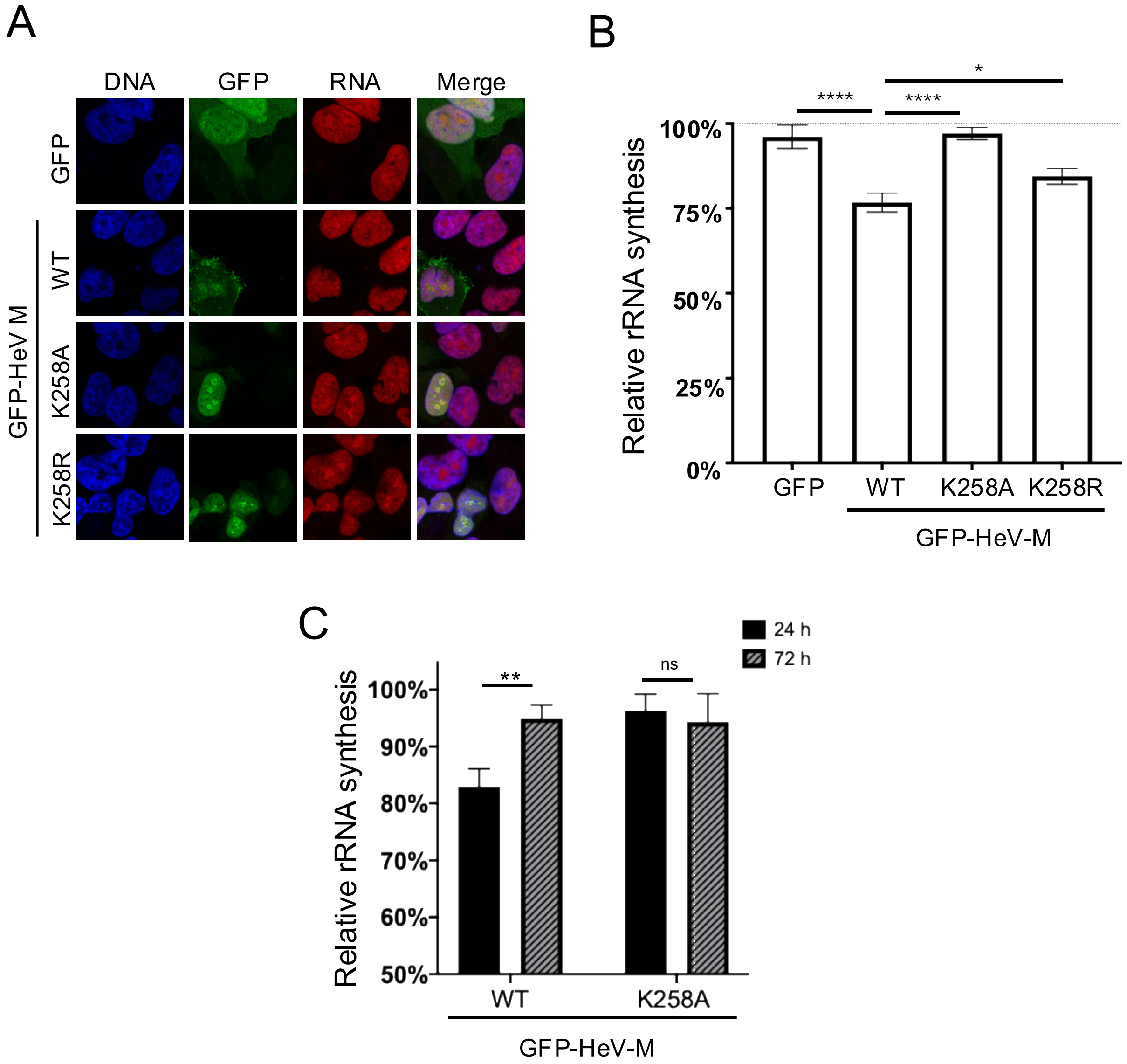

<u>Figure S4.</u> Inhibition of rRNA biogenesis by HeV M protein correlates with dynamic localization to the FC-DFC. (A) HeLa cells were transfected to express the indicated GFP-fused proteins, followed by addition of EU reagent at 23 h post-transfection (p.t.) for 1 h. Cells were then fixed at 24 h p.t., labeled to detect nascent RNA (EU fluorescence) and DNA (Hoechst 33342), and imaged by confocal laser scanning microscopy (CLSM). (B) Images such as those from (A) were used to quantify EU fluorescence in the nucleoli of GFP-positive cells, relative to non-GFP-expressing cells in the same sample (mean relative EU fluorescence ± S.E.M.; n ≥ 51 cells from two independent experiments). (C) Relative rRNA synthesis for indicated proteins and times p.t. was determined as in (B) (mean relative EU fluorescence ± S.E.M.; n ≥ 22 cells). p < 0.05; **p < 0.001; ***p < 0.0001; ns, not significant.

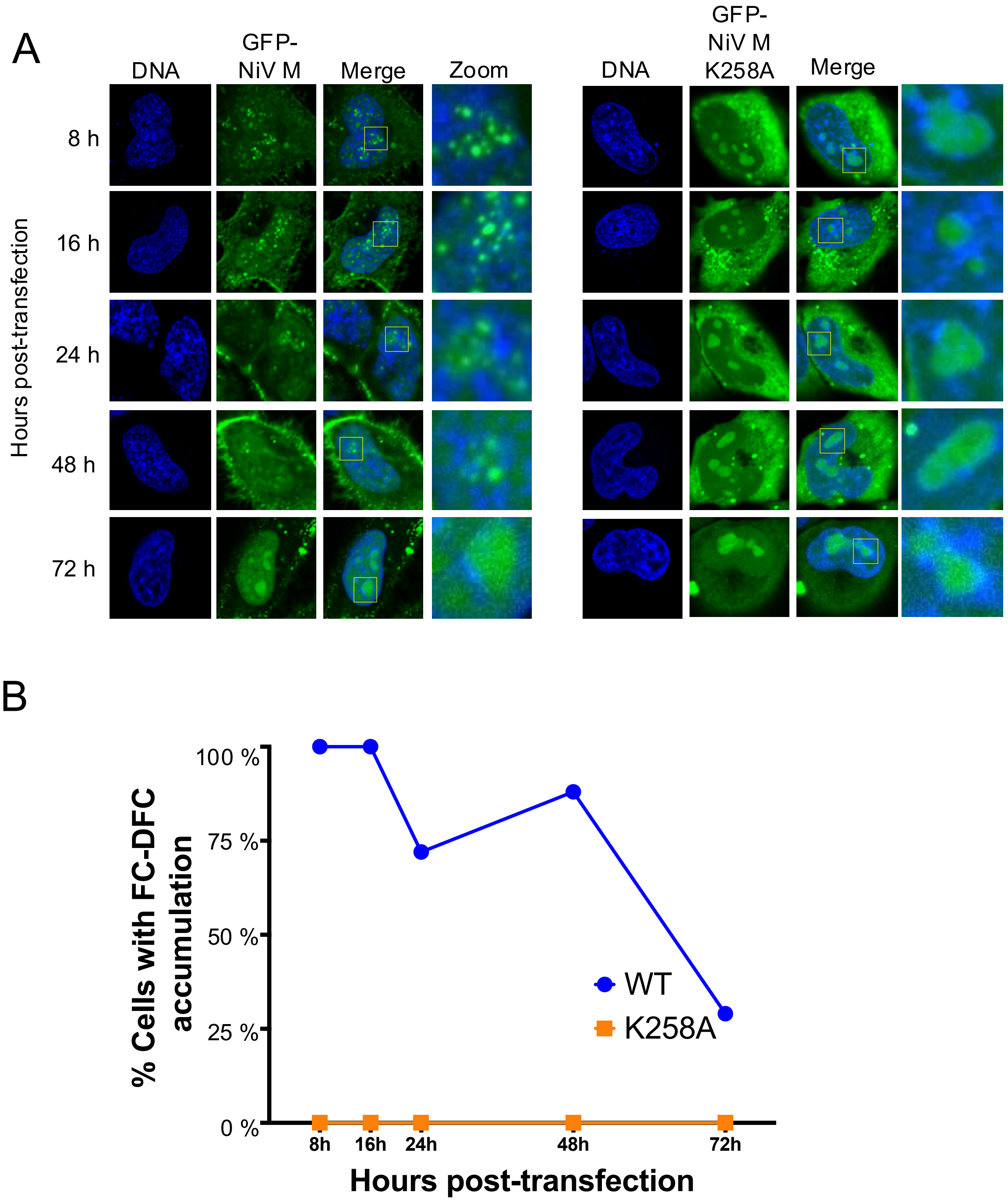

<u>Figure S5.</u> FC-DFC accumulation of NiV M protein decreases over time.
(A) HeLa cells transfected to express GFP-NiV M WT or K258A proteins were analyzed live at 8, 16, 24, 48, and 72 h p.t. by CLSM. Images representative of major phenotypes are shown for each condition, with yellow boxes magnified in the zoom panel. (B) Images such as those in (A) were used to determine the percentage of cells with apparent FC-DFC accumulation. The number of cells analyzed to determine the percentage for each sample is indicated on the graph. n ≥ 18 cells were analysed for each time point.
